# Phenoconversion from Spastic Paraplegia to ALS/FTD Associated with *CYP7B1* Compound Heterozygous Mutations

**DOI:** 10.3390/genes12121876

**Published:** 2021-11-25

**Authors:** Julian Theuriet, Antoine Pegat, Pascal Leblanc, Sandra Vukusic, Cécile Cazeneuve, Stéphanie Millecamps, Guillaume Banneau, Marine Guillaud-Bataille, Emilien Bernard

**Affiliations:** 1 Centre SLA de Lyon, Hôpital Neurologique P. Wertheimer, Hospices Civils de Lyon, Université de Lyon, 59 Boulevard Pinel, CEDEX, 69677 Bron, France; julian.theuriet@chu-lyon.fr (J.T.); antoine.pegat@chu-lyon.fr (A.P.); 2 Institut NeuroMyoGène, CNRS UMR5310, INSERM U1217, Faculté de Médecine Rockefeller, Université Claude Bernard Lyon I, 8 Avenue Rockefeller, CEDEX 08, 69373 Lyon, France; pascal.leblanc@univ-lyon1.fr; 3 Service de Neurologie, Sclérose en Plaques, Pathologies de la Myéline et Neuro-Inflammation et Fondation Eugène Devic EDMUS Pour la Sclérose en Plaques, Hôpital Neurologique Pierre Wertheimer, Hospices Civils de Lyon, 59 Boulevard Pinel, CEDEX, 69677 Bron, France; sandra.vukusic@chu-lyon.fr; 4 Unité Fonctionnelle de Neurogénétique Moléculaire et Cellulaire, Département de Génétique Médicale, GHU AP-HP, Sorbonne Université, Bâtiment de la Pharmacie Secteur Salpêtrière 47/83, Boulevard de l’Hôpital, CEDEX 13, 75651 Paris, France; cecile.cazeneuve@chu-lyon.fr (C.C.); banneau.g@chu-toulouse.fr (G.B.); marine.guillaudbataille@aphp.fr (M.G.-B.); 5 Institut du Cerveau, ICM, Inserm U1127, CNRS UMR7225, Sorbonne Université, Hôpital Pitié-Salpêtrière, 47 Boulevard de l’Hôpital, CEDEX, 75646 Paris, France; stephanie.millecamps@icm-institute.org

**Keywords:** hereditary spastic paraplegia, amyotrophic lateral sclerosis, SPG5, frontotemporal dementia, ALS/FTD, *CYP7B1*

## Abstract

Biallelic mutations in the *CYP7B1* gene lead to spastic paraplegia-5 (SPG5). We report herein the case of a patient whose clinical symptoms began with progressive lower limb spasticity during childhood, and who secondly developed amyotrophic lateral sclerosis/frontotemporal dementia (ALS/FTD) at the age of 67 years. Hereditary spastic paraplegia (HSP) gene analysis identified the compound heterozygous mutations c.825T>A (pTyr275*) and c.1193C>T (pPro398Leu) in *CYP7B1* gene. No other pathogenic variant in frequent ALS/FTD causative genes was found. The *CYP7B1* gene seems, therefore, to be the third gene associated with the phenoconversion from HSP to ALS, after the recently described *UBQLN2* and *ERLIN2* genes. We therefore expand the phenotype associated with *CYP7B1* biallelic mutations and make an assumption about a link between cholesterol dyshomeostasis and ALS/FTD.

## 1. Introduction

Spastic paraplegia-5 (SPG5) is a rare disease (estimated prevalence of about 1:1,000,000 people [1]) associated with recessive mutations in the *CYP7B1* gene, encoding a cytochrome P450 7a-hydroxylase implicated in cholesterol metabolism [2]. Loss of function of this gene leads to accumulation of the neurotoxic 27-hydroxycholesterol and 25-hydroxycholesterol [3] and to progressive neurodegeneration of the corticospinal tract, particularly in the lower limbs. Patients usually present with the classic phenotype of progressive and slowly progressing spastic paraplegia, starting with lower limb predominant weakness and upper motor neuron signs during childhood. In SPG5, cognitive and behavioral functions [2] and the peripheral nervous system are usually spared [4]. Conversely, amyotrophic lateral sclerosis (ALS) is a rapidly evolving neurodegenerative disease involving both upper and lower motor neurons, leading to death after a mean of 3 years, frequently from respiratory failure [5]. Its prevalence approaches 3:100,000 people [5]. ALS is also associated with fronto-temporal dementia (ALS/FTD) in 10% of cases, which causes progressive cognitive and behavioral impairment [5]. Hereditary spastic paraplegia (HSP) and ALS can share a common genetic background, as seen, for example, with the *KIF5A* gene, although the mutations are not located in the same regions of this gene [6]. Recently, two genes, *UBQLN2* and *ERLIN2*, have been associated with the phenoconversion from HSP to ALS, i.e., the sequential occurrence of both diseases in a same individual, raising questions about a pathophysiological link between these conditions [7,8,9]. We report herein for the first time the occurrence of fatal ALS/FTD in a 67-year-old patient suffering from HSP since childhood, associated with pathogenic compound heterozygous mutations of the *CYP7B1* gene, broadening the genetic landscape of this new neurological syndrome.

## 2. Case Report

### 2.1. Proband

The patient developed a progressive lower limb spasticity during childhood associated with pyramidal signs on the lower limbs; this led to the diagnosis of HSP in early adulthood. He reported a progressive deterioration of spasticity, leading to the implantation of intrathecal baclofen pump at the age of 56 years and to the need for a walking aid at the age of 66 years.

When he was 67 years of age, he was referred to our neurology department because of 6-month history of upper limb weakness, dysphonia, and dysphagia. Clinical examination found diffuse muscle atrophy, affecting calves, thenar hand muscles (split hand sign), triceps, and deltoids. Profuse fasciculations were noted on the triceps and interosseous muscles. Deep tendon reflexes were increased in the four limbs. Bilateral Babinski sign and a bilateral Hoffman sign were noted. Muscle strength was 4/5 on proximal and distal upper limbs muscles, according to the Medical Research Council scale, and 3/5 on psoas, 4/5 on tibialis anterior and gastrocnemius muscles. Brain magnetic resonance imaging (MRI) was normal. Spinal cord MRI showed degenerative disk disease without nervous conflict. Needle electromyography found chronic and active denervation in proximal and distal muscles of the four limbs, with fibrillation potentials, positive sharp waves, and fasciculations. Analysis of the cerebrospinal fluid was normal (no white cell, no red cell, normal protein level [0.28 g/L], no oligoclonal band, sterile culture). Human immunodeficiency virus, hepatitis B virus, hepatitis C virus, human T-cell lymphotropic virus type 1, Lyme disease, and syphilis serologies were negative in the blood. Pulmonary function tests found a normal forced vital capacity (FCV; 86%). Diagnosis of ALS was made according to Gold Coast criteria [10]; riluzole 50 mg twice a day was introduced.

He became wheelchair-bound at the age of 69 years. The same year, cognitive disorders including executive functions impairment and behavior disorders such as socially inappropriate behavior, apathy, and binge eating were noted, associated with diffuse cortical atrophy on MRI, suggesting a probable associated fronto-temporal dementia (FTD) according to the Rascovsky criteria [11]. At the age of 70 years, his pulmonary function declined (54% FCV). Non-invasive ventilation was not initiated because of major cognitive disorders. He finally died from a pulmonary infection the same year.

### 2.2. Parents

The parents were not consanguineous and asymptomatic; his only daughter was also asymptomatic.

### 2.3. Molecular Genetic Analyses

After obtaining written informed consent, ethylenediaminetetraacetic acid (EDTA) blood samples were obtained from the patient and his daughter. DNA was isolated using the QIA symphony DSP DNA Midi kit (QIAGEN GmbH, Hilden, Germany).

ALS/FTD and HSP genetic analysis was performed at the Pitié-Salpêtrière university hospital (Paris, France) by targeted next-generation sequencing, using SeqCap EZ library technology (Roche-NimbleGen, Madison, WI, USA), and Illumina sequencing (Illumina Inc., San Diego, CA, USA) on a Miseq platform. The ALS/FTD panel included exons and flanking regions of *ALS2*. *ANG*. *CHMP2B*. *CSF1R*. *DCTN1*. *FIG4*. *FUS*. *GRN*. *HNRNPA2B1*. *MAPT*. *OPTN*. *SETX*. *SOD1*. *SPART/SPG20*. *SQSTM1*. *TARDBP*. *TBK1*. *TREM2*. *UBQLN2*. *VAPB*. and *VCP*. The patient was screened for abnormal repeat expansion in the *C9ORF72* gene by copy number dosage of hexanucleotides by fluorescent polymerase chain reaction (PCR) and repeat-primed PCR. The HSP panel included exons and flanking regions of *ABCD1*. *ALDH18A1*. *ALS2*. *AMPD2*. *AP4B1*. *AP4E1*. *AP4M1*. *AP4S1*. *AP5Z1*. *ARL6IP1*. *ARSI*. *ATL1*. *ATP13A2*. *B4GALNT1*. *BICD2*. *BSCL2*. *C12orf65*. *C19orf12*. *CAPN1*. *CCT5*. *CPT1C*. *CYP2U1*. *CYP7B1*. *DDHD1*. *DDHD2*. *ENTPD1*. *ERLIN1*. *ERLIN2*. *FA2H*. *FBXO7*. *FLRT1*. *GAD1*. *GBA2*. *GJA1*. *GJC2*. *HSPD1*. *KIF1A*. *KIF1C*. *KIF5A*. *L1CAM*. *LYST*. *MAG*. *MARS*. *NIPA1*. *NT5C2*. *PGAP1*. *PLP1*. *PNPLA6*. *PSEN1*. *RAB3GAP2*. *REEP1*. *REEP2*. *RTN2*. *SACS*. *SAMHD1*. *SETX*. *SLC16A2*. *SLC33A1*. *SPART*. *SPAST*. *SPG11*. *SPG21*. *SPG7*. *TECPR2*. *TFG*. *USP8*. *VCP*. *VPS37A*. *WASHC5*. *WDR48*. *ZFR*. and *ZFYVE26*.

**Figure 1:**
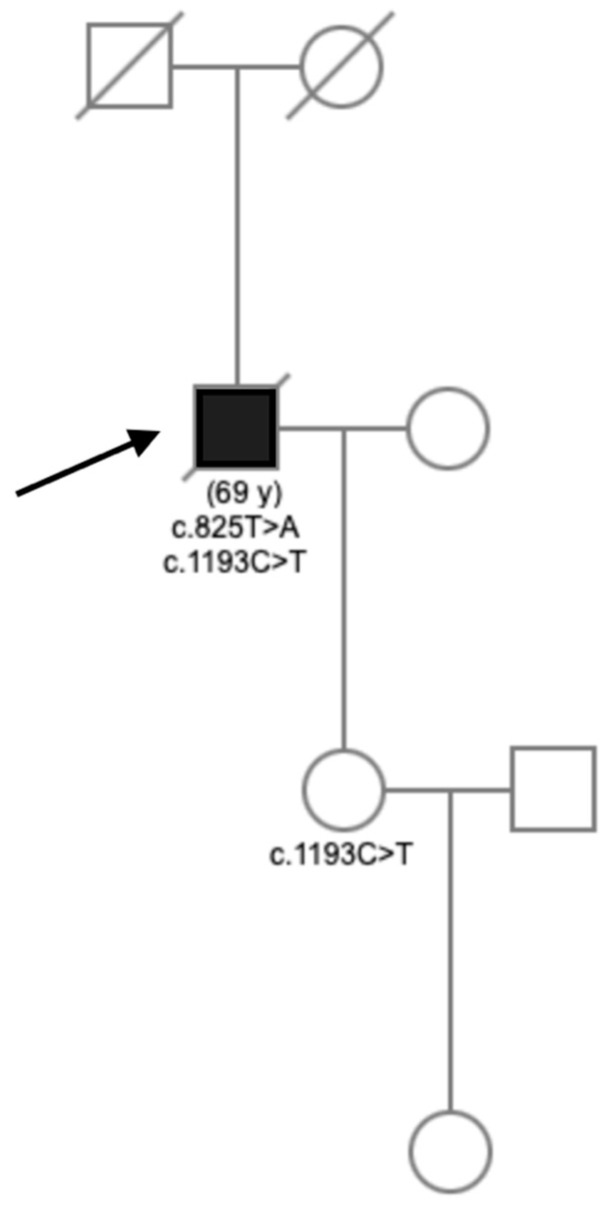
Pedigree of the patient carrying the c.825T>A and c.1193C>T mutations. Index case is indicated by an arrow. When available, age of death (in years, y) is indicated in brackets. The genotypes are indicated for the cases with available DNA. Black fill: phenoconverter from spastic paraplegia to amyotrophic lateral sclerosis with fronto-temporal dementia.

No mutation in the 21 ALS/FTD-related genes was identified. HSP panel analysis identified the following *CYP7B1* compound heterozygous gene variants: c.825T>A (pTyr275*) and c.1193C>T (pPro398Leu). Both variants have been reported in SPG5 patients with altered plasma oxysterols levels [12]. Furthermore, a segregation study confirmed that these variants were situated in trans, due to the absence of the c.825T>A variant, and the heterozygosity for the c.1193C>T variant in the daughter (Figure 1). The c.825T>A and the c.1193C>T variants were considered as pathogenic (class 5) and probably pathogenic (class 4), respectively, according to the American College of Medical Genetics and Genomics criteria [13].

## 3. Discussion

Recently, two genes have been identified to be associated with a phenotype characterized by the development of HSP during childhood or early adulthood, followed by the development of rapidly progressive ALS in adulthood. The first is the dominant X-linked *UBQLN2* ALS-related gene, described in a patient who presented with lower limb upper motor neuron signs that led to the diagnosis of HSP at the age of 35 years, and who secondly developed an ALS at the age of 45 years characterized by the diffusion of upper motor neuron signs to bulbar and cervical regions resulting in tetraplegia within 12 months, associated with dysarthria and dysphagia; transmission was X-linked [7]. The second is the *ERLIN2* gene, a pathogenic variant of which was identified in eight patients belonging to two different families (6 patients), and two sporadic cases; transmission was dominant in one family and recessive in the other [8].

To our knowledge, the patient presented herein is the first case of SPG5 who secondly developed ALS/FTD. The causal link between *CYP7B1* variants and such a phenotype is supported by the previous descriptions of the same syndrome with the two other HSP-causing genes, *UBQLN2* and *ERLIN2*, and the absence of other mutation in any other known ALS genes. Furthermore, given the respective prevalence of SPG5 and ALS/FTD in the general population, a chance association seems unlikely. Although speculative, we can note that *ERLIN2* is involved in the regulation of cholesterol homeostasis too [14], which can underline a plausible common pathophysiology, and is in line with the current literature on the possible link between oxysterols and the pathophysiology of ALS [15,16]. These considerations are of utmost importance given the potential treatable nature of SPG5 with cholesterol lowering drugs [2]. More generally, the present report further substantiates the recently recognized syndrome of phenoconversion from HSP to ALS or ALS/FTD.

## 4. Conclusions

Although definitive conclusions cannot be drawn from a single case, this report suggests that pathogenic mutations in *CYP7B1* gene may be responsible of the phenoconversion from HSP to ALS/FTD. Comprehensive genetic studies of additional cases worldwide are needed to unveil the pathophysiology of this syndrome.

## Data Availability

Not applicable.
